# Development of Novel Tetrapyrrole Structure Photosensitizers for Cancer Photodynamic Therapy

**DOI:** 10.3390/bioengineering9020082

**Published:** 2022-02-19

**Authors:** Natalia Plekhova, Olga Shevchenko, Oksana Korshunova, Aleksandra Stepanyugina, Ivan Tananaev, Vladimir Apanasevich

**Affiliations:** 1 Central Research Laboratory of Pacific State Medical, University of the Ministry of Health of Russia, 690002 Vladivostok, Russia; tarakovaolga@gmail.com (O.S.); farmaoks@yandex.ru (O.K.); stepanyugina@gmail.com (A.S.); oncolog222@gmail.com (V.A.); 2 Academic Department of Nuclear, Technologies of Far Eastern Federal University, 10 Ajax Bay, 690922 Vladivostok, Russia; tananaev.ig@dvfu.ru

**Keywords:** cancer, photodynamic therapy, properties, photosensitizers, porphyrins, tetrapyrrole structure

## Abstract

The effectiveness of photodynamic therapy (PDT) is based on the triad effects of photosensitizer (PS), molecular oxygen and visible light on malignant tumors. Such complex induces a multifactorial manner including reactive-oxygen-species-mediated damage and the killing of cells, vasculature damage of the tumor, and activation of the organism immunity. The effectiveness of PDT depends on the properties of photosensitizing drugs, their selectivity, enhanced photoproduction of reactive particles, absorption in the near infrared spectrum, and drug delivery strategies. Photosensitizers of the tetrapyrrole structure (porphyrins) are widely used in PDT because of their unique diagnostic and therapeutic functions. Nevertheless, the clinical use of the first-generation PS (sodium porfimer and hematoporphyrins) revealed difficulties, such as long-term skin photosensitivity, insufficient penetration into deep-seated tumors and incorrect localization to it. The second generation is based on different approaches of the synthesis and conjugation of porphyrin PS with biomolecules, which made it possible to approach the targeted PDT of tumors. Despite the fact that the development of the second-generation PS started about 30 years ago, these technologies are still in demand and are in intensive development, especially in the direction of improving the process of optimization split linkers responsive to input. Bioconjugation and encapsulation by targeting molecules are among the main strategies for developing of the PS synthesis. A targeted drug delivery system with the effect of increased permeability and retention by tumor cells is one of the ultimate goals of the synthesis of second-generation PS. This review presents porphyrin PS of various generations, discusses factors affecting cellular biodistribution and uptake, and indicates their role as diagnostic and therapeutic (theranostic) agents. New complexes based on porphyrin PS for photoimmunotherapy are presented, where specific antibodies are used that are chemically bound to PS, absorbing light from the near infrared part of the spectrum. Additionally, a two-photon photodynamic approach using third-generation photosensitizers for the treatment of tumors is discussed, which indicates the prospects for the further development of a promising method antitumor PDT.

## 1. Introduction

A wide-scale research of the causes of mortality in the population marked the epidemiological transition between various types of chronic diseases [1] in 2019. According to the data of this study, the leadership of oncological diseases is noted in economically and socially developed countries, where for this reason twice as many people die as from cardiovascular diseases. Surgical methods, radiation and chemotherapy are traditionally used in the treatment of cancer. It has serious side effects and patients undergoing these procedures acquire various somatic pathologies as a rule. At the moment, the search for alternative regimens that can provide a cure with minimal side effects is relevant, and photodynamic therapy (PDT) is one of them. The destruction of neoplasms by PDT is carried out using multifactorial mechanisms: by direct action on cells, causing their death, necrosis and/or apoptosis [2], influencing on the tumor through vascular damage and depriving it of oxygen and nutrients [3], by stimulating the immune system and inducing a local inflammatory response [4].

PDT for the treating of tumors is based on the ability of photosensitizers (PSs) to selectively accumulate in the tumor tissue and stimulate the production of singlet oxygen and its active radicals by cells under local exposure of irradiation with a specific wavelength. To enhance the antitumor effect of PDT and reduce invasiveness to normal tissues, it is necessary to increase the selectivity of PS accumulation by tumor cells and improve its tumor targeting. Moreover, many types of cancer exist in the deeper layers of the body that are far from surface light radiation, so one of the important characteristics of PS is the ability to absorb energy in the longer wavelength range of light radiation. PSs are subdivided into fluorescent and thermal ones, depending on the changes in structures upon transition to an excited state. Fluorescent PSs can be applied to develop sensitive methods for quantitative analysis of their distribution in cells or tissues, which makes it possible to obtain an image of its accumulation in vivo in animals or patients. Additionally, PSs are classified according to their chemical structure into non-porphyrin and porphyrin (or tetrapyrrole) compounds. Common non-porphyrin structures include compounds based on phenothiazine dyes (analogs of methylene and toluidine blue), cyanines (merocyanine 540), and polycyclic aromatic compounds, including hypericin and hypocrellin. The most famous porphyrin PS, containing tetrapyrrole structures, hemoglobin (HPD), chlorophyll and bacteriochlorophyll, as well as porphyrins (in particular, photofrin), were the first PSs used in the clinical practice [5]. The Q-band of tetrapyrrole PSs is about 630 nm (porphyrins, 633 nm; chlorins, 650 nm; 4,4-difluoro-4-bora-3a, 4a-diaza-s-indacene (BODIPY), 523 nm (in ethanol); H2-phthalocyanines, 680–700 nm in N, N-Dimethylformamide (DMF); Zn-phthalocyanines, 702 nm in DMF.

PSs used in photodynamic therapy are classified according to historical development and conceptual approaches to synthesis into first-, second- and third-generation drugs [6,7,8]. The first generation of PS with a tetrapyrrole structure includes sodium porfimer and hematoporphyrins (HpD). The second generation is synthetic compounds that come off or includes the porphyrins, bacteriochlorins, phthalocyanines, chlorins, benzoporphyrins, curcumin synthesized, and others conjugated with various target molecules. Finally, the third generation represents PSs encapsulated in various carriers with target fragments, which increases their tumor selectivity. This review presents PSs of the porphyrin ranges and the new methods of their synthesis, including the use of nanotechnologies, and also shows the prospects for their application by PDT in oncological diseases.

## 2. The Meaning and Mechanism of Cell Damage during Photodynamic Therapy

**Figure 1:**
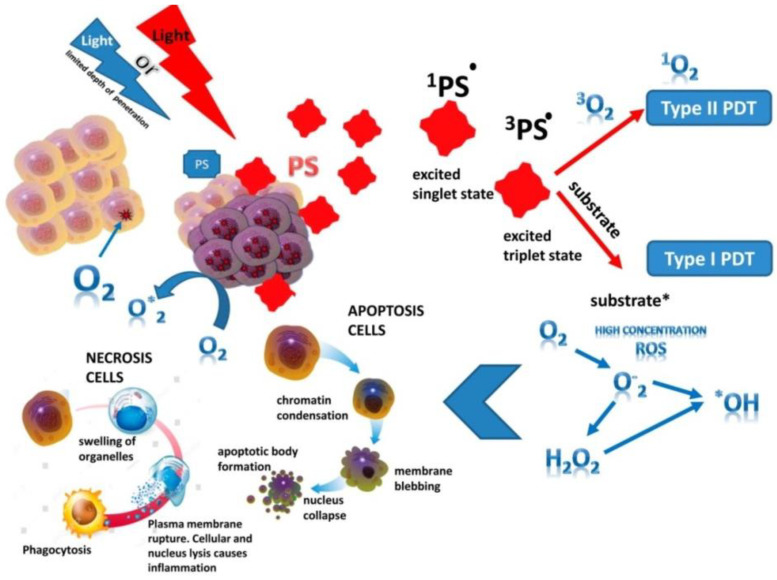
The principle of photodynamic reaction. PS—photosensitizer (*: activated state).

Photoreactions. The PS singlet state is characterized by the presence of two low-energy electrons with opposite spins on the molecular orbital. After absorbing of photons emitted by a specific wave of light, one of the electrons moves to a higher energy orbital while maintaining its spin (the first excited singlet state) (Figure 1). The electron at this state exist for nanoseconds and loses its energy, emitting light (fluorescence) or converting it into heat. The excited singlet state PS is characterized by the fact that the spin of the activated electron is inverted into the triplet state for a relatively long period (from microseconds to milliseconds), in which both electron spins are parallel–intersystem crossing [9].

The excited PS triplet can interact with molecules according to Jablonski by three types of reactions. At I type reactions, the triplet PS can receive an electron from a nearby reducing agent. For example, it is a nicotinamide adenine dinucleotide (NADH) molecule or reduced NAD phosphate (NADPH) in cells. In this case, PS is a radical anion that does not have an additional unpaired electron (PS^−•^). In another case, two triplet PS molecules as radical cation and anion can interact with each other with intermolecular electron transfer. The radical anion forms PS can react with oxygen, which results in electron transfer and the formation of reactive oxygen species (ROS), in particular superoxide anion (Figure 1).

In II type reaction, the PS triplet transfers its energy directly to molecular oxygen and singlet oxygen is formed in an excited state (Figure 1). In this reaction, PSs retain their molecular structure in a multiple photoactivated state. In some cases, one PS molecule can generate 10,000 singlet oxygen molecules. Singlet oxygen, formed during the reactions of type II, is considered the most important molecule responsible for cell damage during PDT [10,11,12]. However, due to the high reactivity and short half-life of singlet oxygen, only the molecules and structures located near the region of its localization are influenced by PS. The least common type of reaction is III, where PS in the triplet state directly reacts with the biomolecule and only they are simultaneously destroyed, without the participation of oxygen. It should be noted that reactions of types I and II can occur simultaneously, and the ratio between them depends on the type of PS used, the concentration of the substrate and oxygen and its active forms (singlet oxygen, O_2_^•^, H_2_O_2_^•^; OH^•^ and NO). The aforementioned ROS are oxidizing agents that can directly react with amino acid residues in proteins, including cysteine, methionine, tyrosine, histidine, and tryptophan. These molecules were found in cells and tissues after PDT [13,14], which indicates of their decisive role in PS-induced cytotoxic effects [15,16]. If PS is not localized in the cell, its photodynamic activity is relatively low. The efficiency of photosensitization is determined by the quantum yield of the formation of the triplet state of oxygen molecules. In this case, the chemical transformation of the PS does not occur, and after the transfer of the excitation energy to molecular oxygen, it returns to the ground stable state, and the whole cycle can be restarted after the absorption of a new quantum of light energy [17].

Antitumor effects of PDT on cells. The phototoxic effect of PS on tumor cells is realized by their direct destruction upon interaction with an organic molecule, which acquires a hydrogen atom or an electron. As a result, a superoxide anion radical is generated in type I photoreaction, or by indirect interaction with molecular oxygen also with the formation of singlet oxygen in photoreaction II type. The ratio of reactions of type I and type II in the tissues of the organism depends on the structure of the PS, the substrate, the concentration of oxygen and the affinity of its binding to the substrate [2,4,15]. Type II reaction prevails during PDT; in this case, singlet oxygen is the main cytotoxic agent inducing biological effects [13]. Intracellular targets of PS are mitochondria, endoplasmic reticulum, lysosomes, the Golgi apparatus, and the plasma membrane of cells. The generation ROS in photodamage of cellular structures leading to cell death (Figure 1). Most PSs are not localized in the nucleus; therefore, PDT does not cause DNA damage, mutations, and carcinogenesis. It is noted that the probability of cell death depends on the lipophilic properties of PS and of the hydrophilic drugs, which is much lower and thus indicates the decisive role of membrane structures in cell damage [18]. So, it has been shown that PS activation with the generation of hydroxyl radicals in the endoplasmic reticulum is a more effective strategy for enhancing the therapeutic effect of PDT than its lysosomal localization with the production of hydrogen peroxide in lysosomes [19]. The most active of the PSs have low toxicity in the dark and high phototoxicity under irradiation [20]. In addition to separating PSs by types of photochemical mechanism (type I or II), they are also distinguished by their properties to localize in cell organelles (lysosomes, endoplasmic reticulum, mitochondria, etc.). So, hydrophobic PSs are localized to a greater extent in mitochondria and endoplasmic reticulum due to the large proportion of lipid bilayers in these organelles, which is much more likely to cause apoptosis. The phototoxic effect of such PSs uses more type I photochemistry and create hydroxyl radicals when surrounded by an aqueous environment, whereas hydrophilic PSs are localized mainly in lysosomes and undergo type II photochemical reactions, inducing the formation of a large amount of singlet oxygen [21]. In 2019, a study by Baptista [22] radically changed the idea that PS are active only when they are localized in the cell. The fundamental role of contact-dependent reactions, which usually cause PS photobleaching and irreversible damage to biological membranes, has been shown. Thus, Mg (II) porphyrazines (MgPzs), which have similar quantum yields of singlet oxygen and side groups with different electron-withdrawing strengths, have different redox properties. The process of their photobleaching is based on the photoinduced detachment of electrons from the surrounding electron-rich molecules (solvent or lipid molecules) and the formation of a radical anion. The photobleaching of MgPzs porphyrazines depends on the degree of lipid unsaturation due to the detachment of electrons from the lipid double bond when incorporated into phospholipid membranes. A high rate of induced leakage in membranes corresponds to a high rate of photobleaching. The results obtained have a major impact on further strategies for the development of new photosensitizers.

In PDT, the death of tumor cells occurs in a programed (apoptosis) or unprogramed (necrosis) way [23]. At high light intensity, tumor cells die through necrosis, which is characterized by vacuolization of the cytoplasm and destruction of the cell membrane (Figure 1). In this case, a local inflammatory reaction occurs in response to the appearance of cellular debris and pro-inflammatory mediators in the extracellular space [24]. Low doses of light during PDT initiate the genetically encoded cell death, or apoptosis [22,25]. During this process, the structure of cells changes, nuclear chromatin condenses, and chromosomal DNA is cleaved into internucleosomal fragments (Figure 1). At this time, cells decrease in size and apoptotic bodies are formed, surrounded by a plasma membrane [26]. This type of cell death does not induce an immune response, since cellular debris does not appear in the extracellular space [22,27]. Apoptosis can turn into necrosis with an excessive decrease in the availability of caspases and the intracellular concentration of adenosine triphosphate (ATP) [28]. Additionally, when apoptosis is disturbed, another mechanism of cell damage is paraptosis with the photodamage of the endoplasmic reticulum during PDT [29]. Paraptosis proceeds independently of the activation and inhibition of caspases, without condensation of chromatin and fragmentation of the nucleus with the vacuolization of the cytoplasm in contrast to apoptosis.

The Photodynamic Processes. The success of PDT is based on PSs’ ability to penetrate into the target tumor with minimal damaging effect on the healthy tissues of organism. In cancer therapy, depending on the localization of the tumor, PS can be administered intravenously, orally, or locally. ROS is generated at the site of the localization of the PS where it is irradiated with a light source of the appropriate wavelength, which indicated the death of cancer cells without affecting healthy tissues. The maximum concentration of PS is reached in tissues after 24–72 h. The direct destruction of tumor cells with the using of PS also leads to the destruction of tumor microvessels, since endothelial cells can concentrate PS, initiating the production ROS that is activated by the appropriate light. The violation of the vascular walls (vascular effect) during PDT leads to the cessation of access to the tumor of oxygen and nutrients, which indicates the long-term effectiveness of such a therapy [24]. The effectiveness of the vascular effect of PDT is achieved by using a short interval between the systemic injection of PS with its localization in the vascular network and the precise effect of radiation on the tumor [27].

In addition to direct and vascular effects, PDT can significantly affect the adaptive immune response by stimulating or suppressing it. Its effectiveness depends on the degree of antitumor immunity induction. So, if the long-term control of a tumor with a combination of direct and vascular effects of PS activates the immune response [30], then the immunosuppressive effect of PDT is mainly associated with reactions to local treatment with a high flow rate and over large areas of irradiation [24]. Changes in tissue integrity and homeostasis with cell necrosis induce an acute inflammatory response due to oxidative damage to the tumor stroma during PDT, initiated by the release of pro-inflammatory mediators, including various cytokines, growth factors, and proteins [24,25]. Innate immune cells (neutrophils, mast cells, macrophages and dendritic cells) at the site of injury phagocytose the breakdown products of cancer cells and provide proteins to the helper CD4^+^ T lymphocytes [31]. The cytotoxic T cells can recognize and specifically destroy tumor cells and can circulate throughout the body for a long time, providing a systemic anti-tumor immune response. After several cycles of absorption, PS can degrade and lose the ability to trigger a photodynamic reaction; in this case, the process of its burnout is called photobleaching [32]. On the other hand, the insufficient penetration depth of laser light (4–8 mm depending on the wavelength), lack of a reliable evidence base, difficulties in planning, dosimetry and monitoring of processes due to the complex interaction of photons with biological tissue are restrictions to the application of PDT [33].

## 3. Porphyrin Photosensitizers for Cancer Photodynamic Therapy

According to Pushpan S.K. et al. [34], an ideal photosensitizer for PDT should have the following properties: (a) be available in pure form with a known chemical composition; (b) be synthesized from available precursors with easy reproduction; (c) have a high quantum yield of singlet oxygen (Φ_Δ_); (d) possess strong absorption in the red region of the visible spectrum (680–800 nm) with a high extinction coefficient (ε_max_), for example, 50,000–100,000 M^−1^ cm^−1^; (e) efficiently accumulate in tumor tissue and exhibit low toxicity in the dark, characteristic of both PS and its metabolites; (f) be stable and soluble in tissue fluids of the body and be easily delivered to the body by injection or other methods; and (g) be rapidly excreted from the body after the completion of the treatment.

The first generation of PS with a tetrapyrrole structure includes sodium porfimer and hematoporphyrins (HpD). In order to increase the ability to absorb light in the long-wavelength spectral region and achieve high activity in relation to ROS production, the PS of the first generation was modified. The second generation of PSs is synthetic compounds, including modified derivatives of porphyrins, chlorins, bacteriochlorins, and phthalocyanines and others conjugated with various target molecules. They have an enhanced ability of absorption in the visible and near-infrared electromagnetic spectrum, a high quantum yield of singlet oxygen, and a more predictable dose–response interpretation and therefore have a stronger effect on the tumor. Currently, the best strategy for achieving the high selectivity of PS for certain tumor areas is their combination with biomolecules, antibodies, proteins and carbohydrates [31,35]. Finally, third-generation PSs represent PSs encapsulated in various carriers, which increases their tumor selectivity. For this type of conjugation, nanoparticles of gold, silica, quantum dots, carbon nanotubes and other molecules can be used as carriers [36,37].

Second generation photosensitizers. Currently, the search for new PSs with improved therapeutic properties that absorb light in the near infrared region in the 720–850 nm range continues. These compounds exhibit the ability to activate a high quantum yield of singlet oxygen generation in tissues. In 1995, Tsukagoshi proposed the use of sodium porfimer (Photofrin II) for PDT followed by laser irradiation as a new cancer treatment method [38]. The tumor selectivity of this compound is due to its high affinity for low density lipoproteins (LDLs). It is known that in cancer tissues the expression of LDL receptors is increased, which determines the success of using Photofrin II for PDT [39]. It has been shown that PS is localized in cancer cells on the membranes of mitochondria, endoplasmic reticulum and the Golgi complex [40]. The irradiation of sodium porphimer with a wavelength of 630 nm in tumor cells induces the production of highly active excited singlet oxygen (^1^O_2_) with the release of cytochrome c from mitochondria, followed by the initiation of apoptosis, whereas a modified complex compound of photofrin II with the addition of picolylamine groups and zinc is capable of increasing the production of singlet oxygen many times compared to sodium porphimer [41]. Kano and colleagues presented a new approach to the synthesis of PS. The combination of Photofrin II with polyethylene glycol-poly-lysine (L-lysine) (PLL-g-PEG) enhances the localization of PS in the tumor [42]. In this case, photofrin II is tightly bound to PLL-g-PEG through ionic and hydrophobic interactions, which provides its better anti-cancer efficacy compared to the parent compound.

New analogues of porphyrins were synthesized using pyrrole, various aldehydes, and propionic acid [43]. The evaluation of the photophysical characteristics of these compounds using spectral analysis methods (IR, NMR and mass spectroscopy) showed that the period and quantum yield of fluorescence are not constant due to the presence of a change in the nature of the electrons recoil. The efficiency of singlet oxygen generation by each synthesized porphyrin was recorded through the photooxidation of 9,10-dimethylantacene. It was shown that the synthesized analogs of porphyrins are more characterized by electron-acceptor properties than the reference 5,10,15,20-tetraphenylporphyrin (H_2_TPP). The values of the singlet oxygen quantum yield of the synthesized compounds varied from 0.52 to 0.66, which indicated their photostability, efficiency, and suitability for PDT.

Among the various methods of synthesis, the most widespread is the conjugation of PS with carbohydrate molecules, antibodies, or subcellular target peptides that penetrate the cell. The intensive development of methods for covalent conjugation of the binding of porphyrin units to various bio-macromolecules oligonucleotides (ONs), peptides and antibodies has been carried out since 2002 [44]. An extensive number of different ways of conjugation of PS with biomolecules, such as amide, isothiocyanate, maleimide, SNAP-Tag and «Click», are described [45]. The most commonly used reactions for the synthesis of the PS-peptide moiety are based on the formation of an amide bond with the activation of carboxylic acids. In this way, the commercial preparation Foscan^®^ was obtained by reduction of diimide in 5,10,15,20-Tetrakis(3-hydroxyphenyl)chlorin (mTHPC). In addition, the reaction of azide-alkyne Huisgen cycloaddition, SN_2_-alkylation and Michael reaction for thiol-containing peptides [46] are used.

**Figure 2:**
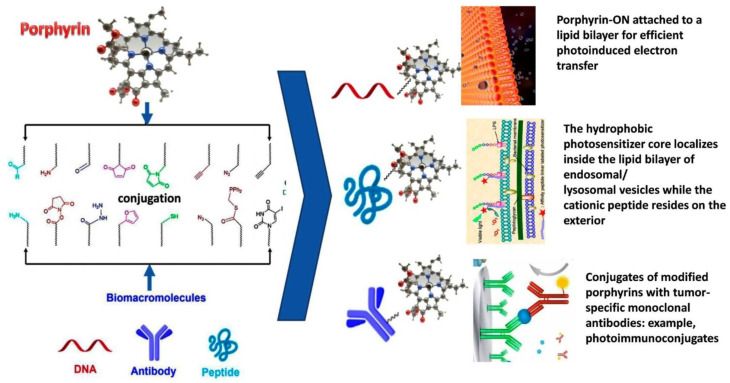
Schematic of the attachment of porphyrins to biomacromolecules using functional groups or bio-orthogonal pairs and some applications of the conjugates.

Molecules that are used to attach to porphyrins contain many functional groups (for example, amines and thiols) (Figure 2). Their reactivity is used for selective conjugation with PS.

Since nucleic acids and peptide systems differ in structure, connectivity, stability, solubility, location and availability of functional groups, there are an individual synthetic approaches to the conjugation of porphyrins with each type of biomacromolecules that are being developed. Moreover, such conjugates have various applications, for example, porphyrins associated with DNA (especially cationic porphyrinoids) are used to detect specific secondary structures of nucleic acids, staining nuclei and as antimicrobial chemotherapeutic agents [47]. Porphyrins can also attach to biomolecules using bio-orthogonal chemistry methods as ON conjugation (attachment of porphyrinoids to sugar ring, attachment of porphyrinoids to nucleobase moiety, porphyrinoid phosphoramidites without nucleoside) or by post-synthesis modifications and subsequent reactions (such as amide combinations, hydrazide-carbonyl reactions, and others). Highly selective bio-orthogonal reactions are designed for individual targets in order to avoid unwanted reactions with other biological/chemical objects present in living cells/systems with certain physiological conditions (ambient temperature and pressure, neutral pH and aquatic environment). Highly selective bio-orthogonal reactions should be kinetically, thermodynamically and metabolically stable and in their course should not form molecules toxic to living systems [48]. The use of ON-porphyrin systems for clinical translational applications is still limited; only their use as antimicrobial biomaterials and site-specific cleavage of nucleic acids has been reported.

The development of new conjugation systems using the carboxylic acid functional group of carbodiimide implies the activation of porphyrin by creating a monomer followed by oligomerization [49,50,51]. Since α-polypeptides can form certain secondary structures (especially α-helices), multiple porphyrin units serve as light-capturing moieties, and a helical peptide conformation can align porphyrins, facilitating exciton migration [51]. Regarding the charge separation step, a supramolecular strategy is used in which metalated porphyrins can also be hosts for fulleropyrrolidines (carrying pyridine, PyC60 or imidazole, ImC60), which are well known for their electron-withdrawing ability [52]. In addition to activation of carboxylic acids in situ, esters of carboxylic acids are also used [53].

The research area of porphyrin–antibody conjugates (photoimmunoconjugates) is rapidly developing, the beginning of which was determined in the 1980s–1990s [54,55]. For the first time, hematoporphyrin was conjugated through the carboxyl moiety to the lysine side chain of a monoclonal antibody against a protein (mAb) secreted by M-1 myosarcoma cells [54]. The conjugate mAb-M1-hematoporphyrin at a low dose (0.268 mg/kg of body weight) significantly increases the efficacy of treating myosarcoma compared to a high dose (2.5–5.0 mg/kg body weight) of hematoporphyrin. Additionally, hematoporphyrin was conjugated to a mAb against a specific antigen associated with leukemia (CAMAL-1). After irradiation with a red light a 620 nm laser, this conjugate is capable of effectively killing tumor cells. Similar methods for conjugating PS with antibodies have been developed for the treatment of gastric and lung cancers [56,57].

In 1993, a benzoporphyrin derivative (BPD) was conjugated to with a tumor-specific antibody against epidermal growth factor receptor (anti-EGFr), where binding through thiolmaleimide was used [58]. In an experimental modeling of adenocarcinoma, 80% of animals (Syrian golden hamsters) compared with control groups (treated with light alone or nonspecific antibody-porphyrin conjugates) showed tumor necrosis and did not become sick for 1 month [59,60]. First, monoethylenediamine chlorin e6 was loaded into the polyglutamic acid backbone by means of carbodiimide coupling, followed by hydrazide modification of the carboxylic acid PGA. The chlorin-PGA-hydrazide reacted with the functionalized aldehyde mAb to form a hydrazone bond. For the stability of the photoimmunoconjugate, a reduction reaction with sodium cyanoborohydride was performed. Various polymer linkers (polyvinyl alcohol, polyglutamic acid, dextran and polylysine) and modification of commercially available PSs with carboxyl have also been used to target tumor cells [61,62]. These polymer linkers are able to improve the physicochemical properties of photoimmunoconjugates, but there is a nonspecific and random distribution of photosensitizers on antibodies.

A group of researchers led by Boyle are investigating porphyrin conjugates with antibodies [63]. Thus, the conjugated 5-(4-isothiocyanatophenyl)-10,15,20-tri-(3,5-dihydroxyphenyl) porphyrin and 5-(4-isothiocyantophenyl)-10,15,20-tris-(4-Nmethylpyridiniumyl) porphyrin to murine mAbs 35A7 and FSP 77 via amine-isothiocyanate coupling mAb 35A7 recognizes carcinoembryonic antigen (CEA), which is overexpressed in colon adenocarcinomas, and mAb FSP 77 recognizes the extracellular domain of the erb-B2 receptor, which is overexpressed in ovarian and breast cancers. In 2010, the same group of researchers conjugated isothiocyanate-functionalized cationic porphyrins with tumor-specific monoclonal antibodies, anti-CD104, anti-CD146 and anti-CD326 [64]. These conjugates showed the same efficacy as the commercial agent Photofrin in shrinking human LoVo tumors in mice at much lower doses (10 nmol/kg versus 8.3 μmol/kg).

A series of conjugates of modified meso-tri(4-pyridyl)-mono-(4-carboxyphenyl) porphyrin with lysed amines, serum albumin and anti-CD104 and anti-Caf mAbs were produced [65]. UV-visible spectroscopy was used to assess the degree of labeling of porphyrins (DOL). These studies indicated that the highest DOL was obtained with an initial porphyrin-to-protein molar ratio of 30:1 (resulting in ~1 and ~2 porphyrins attached to the BSA and HSA conjugates, respectively), and for the porphyrin-mAb anti-Caf and the anti-CD104 porphyrin mAb, the highest DOL values were 0.81 and 0.80, respectively. The method of the site-selective conjugation of cysteine and bio-orthogonal chemical compounds was also used to synthesize a bifunctional linker carrying dibromopyridazinedione and cyclic alkyne units with a deformed ring [66].

Studies moved in the direction of developing PS with properties not to have a damaging effect on surrounding healthy tissues and the ability to target vessels and tumor cells, where overexpression of the somatostatin neuropeptide receptor (sST2), is noted. Kashontsakova and co-workers synthesized two Ce6 chlorin derivatives, Ce6-K3- [Tyr3]-octreotide and Ce6-[Tyr3]-octreotide-K3-[Tyr3]-octreotiwde for PDT treatment using human erythroleukemic cells K562 [59]. It was demonstrated that the first derivative exhibits better anti-cancer properties than the second, due to the difference in hydrophobicity.

Newly synthesized complexes of chlorin (Ce6) with glucose (G-chlorin) are of interest as promising PS conjugates for PDT. It was shown that the antitumor effect of β-glucose-conjugated Ce6 (β-M-Ce6) on human glioblastoma cells U251 in terms of the degree of effect was similar to the complex with glucose β-G-Ce6 and 1000 times higher than the effect of the first-generation PS of talaporphin sodium (TS) [67]. Thus, the uptake of β-M-Ce6 and distribution among organelles of U251 cells, such as the Golgi apparatus, mitochondria, and lysosomes, proceeded much faster than TS. Another conjugated oligosaccharide-conjugated chlorin (O-chlorin) is highly soluble in water and accumulates in the lysosomes of tumor cells [24]. In vivo, O-chlorin showed a better cytotoxic effect in PDT and photodynamic diagnosis (PDD) compared to TS. O-chlorin is a promising candidate for a new generation of bifunctional photosensitizers for both PDT and PDD.

Metalloporphyrin compounds should also be noted as promising for the development of new generations of PS. A series of metalloporphyrin-indomethacin conjugates linked by polyethylene glycol chains have been synthesized and characterized [68]. Singlet oxygen production of conjugates was assessed using 2′, 7′-dichlorofluorescein (DCFH). Compared to porphyrin, its metal complexes exhibited a higher quantum yield of singlet oxygen (^1^O_2_). The low cytotoxicity of these conjugates was revealed on HeLa cells in dark conditions. After irradiation, platinized porphyrin (PtPor) showed the highest therapeutic activity among all other conjugates. A similar effect was exhibited by related porphyrin complexes through the connection of its units with alkyl chains and coordination with palladium molecules (Pd-Monopor, Pd-Dipor and Pd-Tripor) [69]. The ROS generation of the six porphyrin compounds was higher than for free porphyrin, probably due to the heavy atom effect. The efficiency of ROS generation increased with a number of porphyrin units in the structure of PS: Pd-Tripor Tripor Dipor Pd-Monopor Pd-Dipo r Monopor. The low cytotoxicity of these complexes in the dark was shown, which indicates their good biocompatibility. The complex of porphyrins with palladium exhibited a higher cytotoxic activity against tumor cells compared to free base porphyrin under light irradiation. Subcellular localization of these complexes was observed in the lysosomes of cancer cells.

**Figure 3:**
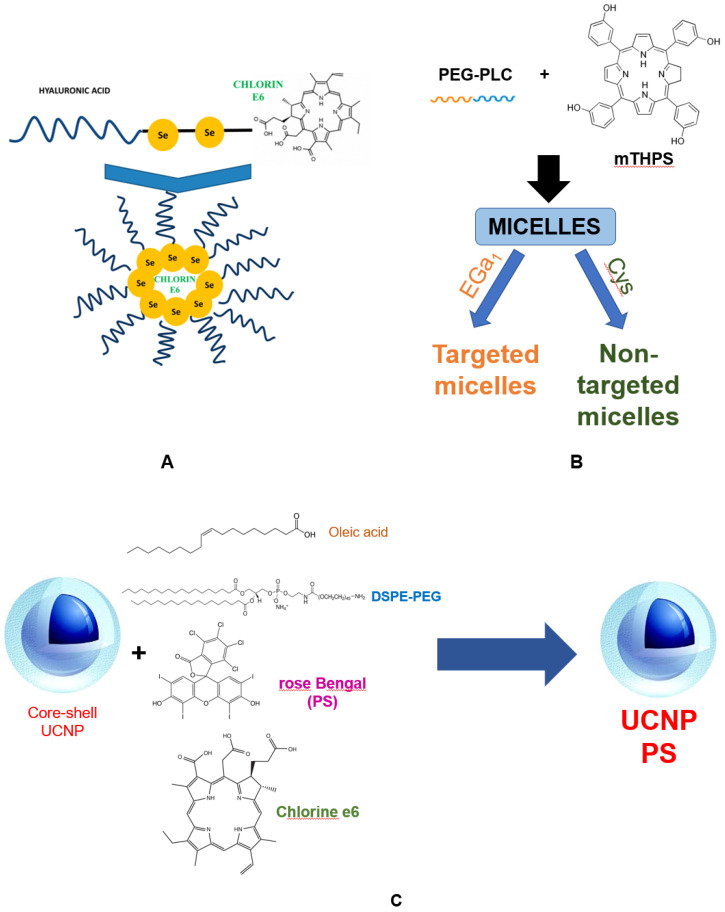
General schemes for the synthesis of PS nanoparticles: (**A**)—Scheme of self-assembling HA-sese-Ce6 micelles, redox sensitive Ce6 release, and positive feedback loop that triggers more Chlorin e6 release; (**B**)—Scheme of upconversion nanoparticles a core-shell structure contain PS Chlorin e6 and Rose Bengal UCNPs; (**С**)—Scheme of зreparation of polymeric micelles conjugated with EGa1 (Targeted) or Cys (Nontar-geted).

Photosensitizers of a new generation. At the moment, the development of new generations of PS is being carried out using the strategy of encapsulating PS in liposomes, micelles, metal frameworks, as well as with the design of nanoparticles as carriers [70]. The main goals in the development of such PS are to reduce the adverse effects on healthy cells surrounding the tumor, and to improve the pharmacokinetics and tumor-specific accumulation of these PS by stimulating bioconjugation with the targeting fragment. The immobilization of target fragments on PS, such as antigen receptors, provides specific binding to tumor cells and their destruction using a photodynamic protocol without damaging normal cells. Since the depth of light penetration into tissues is determined by an increase in wavelength, porphyrins with strong absorption in the red region of the spectrum (chlorins, bacteriochlorins, and phthalocyanines) are promising for designing a new generation of PSs [71]. Chlorin e6 (Ce6) is one of the demanded compounds in the design of a new-generation PS due to its low toxicity in the dark and excellent anticancer properties under irradiation. A Ce6-conjugated amphiphilic polymer poly(ethyleneglycol)-poly(d, l-lactide) (mPEG-PLA-Ce6) was developed, which self-assembles to form stable nanoparticles [72]. The nanoparticles were characterized by particle size, zera potential, and singlet oxygen generation (^1^O_2_). In monolayer and three-dimensional spheroids of human lung adenocarcinoma cells, ^1^O_2_ generation upon exposure to mPEG-PLA-Ce6 was significantly higher than for f free Ce6. The nanoparticles showed increased cell internalization and phototoxicity in these cells, indicating the potential of mPEG-PLA-Ce6 for PDT of solid tumors. The using of diselenide (Se–Se) bonds in the complex of Ce6 with hyaluronic acid (HA) made it possible to create a self-assembling micelle HA–Se–Se–Ce6, which targets the receptors of the differentiation cluster 44 (CD44) overexpressed on cancer cells (Figure 3). After the separation of the micelle in the intracellular redox environment, PS is released from the nucleus. The high targeted internalization of HA–Se–Se–Ce6 cells by orthotopic mammary fat pad tumor model was shown.

The cytotoxicity to cells was also high, indicating an original and simple strategy to improve the efficiency of PDT. A similar structure for immunogenic phototherapy «nucleus-сore-shell», including gold nanoparticles coated with manganese dioxide and hyaluronic acid, was developed for the targeted delivery to colorectal cancer cells to enhance oxygenation. These nanoparticles in the tumor generated a large amount of ROS upon IR irradiation, and also induced immunogenic cell death with the release of associated molecular structures, which promoted the maturation of dendritic cells. These potent antigen presenting cells (APCs) are effective in further enhancing systemic anti-tumor immunity against progressive tumors. The results of in vivo experiments have shown that nanoparticles not only have the ability to target a tumor, but also produce enough oxygen in situ. In addition, APC-mediated immunogenic PDT with increased oxygen content effectively suppressed tumor growth and recurrence.

Another targeted delivery of Ce6 to tumors combines hemotherapy (CT), photodynamic therapy and photothermal therapy (PTT), using reduced graphene oxide (rGO) with MnO_2_ as a carrier for Ce6, and doxorubicin (DOX) as chemotherapy drugs or cisplatin [73]. On the surface of such particles, a ligand targeting HA is conjugated. Since the tumor exhibits extreme pathological hypoxia, doping with MnO_2_ catalyzes the decomposition of H_2_O_2_ into oxygen, while Ce6 enhances the formation of ROS after laser irradiation at 635 nm.

The synthesis of upconversion nanoparticles (UCNPs), the so-called nanoparticles, which can be excited by near-infrared light, belongs to the modern field of technologies for engineering of new PSs for theranostics. UCNPs with a core–shell structure (NaYF4: Yb, Er, Nd@NaYF4:Yb, Nd) were synthesized with irradiation at a wavelength of 808 nm as an excitation source [74]. This wavelength is preferable to the commonly used 980 nm with the effect of overheating the cells. Er-doped UCNP nanoprobes emitting at two wavelengths contain Chlorin e6 and Rose Bengal PSs that increase the efficiency of PDT. Each Ce6 and Rose Bengal photosensitizer in UCNP can absorb red and green wave energy, respectively, to form ROS. The generation of ROS and immunogenic apoptosis of tumor cells under the action of such a complex with two PSs were significantly higher than under the action of one.

From the point of view of theranostics, it is interesting to develop multifunctional nano-biomaterials with the integration of diagnostic and therapeutic functions. A nanoplatform based on functionalized Cu_3_BiS_3_ nanoparticles was synthesized for combined PDT aimed at tumors [75]. Hydrophobic Cu_3_BiS_3_ NPs are modified with the DSPE-PEG/DSPE-PEG-NH2 complex, conjugated with Ce6 and the target folic acid (FA) ligand. Rationally designed NPs Cu_3_BiS_3_-PEG-(Ce6-Gd^3+^)-FA possess high physiological stability and good biocompatibility. They specifically target tumor cells expressing the FA receptor. Cu_3_BiS_3_-PEG-(Ce6-Gd^3+^)-FA nanoparticles demonstrate effective synergistic photothermal/photodynamic therapy. Ce6 chelated nanoprobes with polyethylene glycol and gadolinium ions (PEG-Ce6-Gd NPs) are synthesized by a self-assembly method [76]. In preclinical studies, these non-toxic PEG-Ce6-Gd nanoprobe significantly increase the phototoxicity of PDT when exposed to laser radiation against cancer cells. The high diagnostic and therapeutic efficacy of PEG-Ce6-Gd NPs was demonstrated using a glioma xenotransplantation model in mice.

Another highly efficient hydrophobic PS mTHPC is prone to aggregation in biological fluids, which leads to a decrease in the generation of reactive oxygen species and therapeutic efficacy. To preserve the properties and delivery of mTHPC, polymer micelles with diameters of 17, 24 and 45 nm based on benzyl-poly (ε-caprolactone)-b-poly (ethylene glycol) were constructed. To enhance the uptake of cancer cells, micelles were conjugated with molecules of epidermal growth factor (EGa1) [77]. The results showed a higher uptake of such micelles by A431 tumor cells expressing the EGFR receptor compared to low-expressing HeLa cells. Moreover, mTHPC loaded into micelles with EGa1 demonstrated four times higher photocytotoxicity on A431 cells compared to micelles without PS. Micelles with d-tocopheryl polyethylene glycol 1000 succinate (TPGS) for encapsulating Ce6 (TPGS-IR820/Ce6) have been designed and endowed with a variety of theranostic properties, including fluorescence imaging, PTT and PDT [78]. Stable micelles have a high singlet oxygen production capacity and remarkable photothermal conversion efficiency. After internalization in cells, a single irradiation of micelles with a NIR laser led to their death. Nanoprobes, including sodium talaporphin and Ce6 conjugated with glucose, have been synthesized [79]. Since cancer cells uptake a significant amount of glucose (Warburg effect), they can be detected by positron emission tomography (PET), which indicates the prospects for their using in theranostics. Similar nanoprobes are used as radio- and fluorescent-labeled antibodies targeting cancer cells in surgery for imaging and resection of tumor lesions [79,80].

Nanoparticles with poly-dopamine (PDA) and modified polyethylene glycol (PDA-PEG/Cur/Ce6) are used as a multifunctional nanocarrier of Ce6 and curcumin (Cur) for combined PDT and cancer radiotherapy [81]. In this nanoparticle, Ce6 initiates the generation of singlet oxygen by near infrared laser irradiation for PDT. It should be noted that Cur acts as a radiosensitizer when exposed to X-rays to enhance radiotherapy. As it has been demonstrated in vitro and in vivo models, the combination of PDT and RT using PDA-PEG/Cur/Ce6 nanoparticles causes significant inhibition of cancer cell growth. This study provides not only a theranostic platform for cancer treatment using polymers, but also demonstrates the potential application of combined clinical therapy.

The photophysical and photosensitizing activity of PS chlorin e6 included in the system of plant phospholipids has been studied [82]. The complex of Ce6 with phospholipid nanoparticles causes a bathochromic shift of the absorption maximum of the Q-band by 14 nm without changing the absorption in the Soret band range. The fluorescence intensity of Ce6 incorporated into phospholipid nanoparticles increased 1.7 times. Under irradiation, Ce6 in phospholipid nanoparticles is capable of generating ROS, as shown by the oxidation of polyunsaturated fatty acids of the phospholipid matrix of the delivery system and reduced l-glutathione. In vivo, it was demonstrated that the new PS nanoform with chlorin e6 accumulates in tumor tissues more than its free form.

Along with Ce6, chlorophyll derivatives are also effective novel tetrapyrrole structure photosensitizers. Using nanotechnology, a liposomal form of PS with encapsulated iron chlorophyllin (Fe-CHL) was synthesized. It was evaluated in terms of efficacy in the PDT of melanoma [83]. Liposome nanoparticles were absorbed by cells endocytosis with predominant accumulation in mitochondria and the nucleus. The cells were found to die by a combination of apoptosis and necrosis. Thus, PDT with a monochromatic red laser of 56.2 J/cm^2^ at a wavelength of 652 nm caused 50% cell death after 48 h of incubation with Fe-CHL encapsulated in liposomes. Therefore, the newly synthesized PS can be used as a potential sustained release delivery system in Fe-CHL-mediated PDT. Chlorophyll conjugated to quantum dots (QDs) is also capable of being excited by resonant fluorescence energy transfer (FRET). The chlorophyllin-copper complex and CdSe/ZnS QDs were encapsulated in biodegradable nanoparticles consisting of a copolymer of lactide with glycolide (PLGA) [84]. Upon excitation of such particles with a wavelength of 365 nm, FRET leads to the formation of ROS, both in an aqueous medium and in cells, thereby confirming the potential of the composition of such nanoparticles for PDT in oncology.

Effective photodynamic therapy involves the using of photosensitizer, molecular oxygen and visible light. PDT is an alternative clinical protocol against localized malignant tumors and other diseases. Traditional strategies for developing PS platforms are based on molecular design, which requires specific modifications to each PS prior to PDT. Thus, PDT has been combined with immunotherapy as a promising treatment option for metastatic cancer. From this point of view, it is desirable to create a common tumor sensitive PDT platform with minimal loss of ROS due to an endogenous antioxidant, usually glutathione. On the other hand, the most important problem of PDT is activated phototoxicity for selective destruction of cancer cells with a low level of damage outside the target and at the same time predicting its effect.

The using of the first-generation PS in PDT revealed clinical difficulties, such as long-term photosensitivity of the skin, insufficient penetration into deep-seated tumors and inaccurate localization. The second generation is based on different approaches to the synthesis and conjugation of PS with biomolecules. It is made it possible to approach targeted PDT of tumors. Antibody-porphyrinoid conjugates are widely used in biomedicine for the targeted PDT in oncology. Direct conjugation of functionalized porphyrinoids with peptides is possible due to the reactive functionality of certain amino acids (amine, thiol, and carboxylic acids). However, due to the many similar functional groups present in peptides, these reactions usually require reasonable protection of functional groups. The protein component provides solubility and minimizes PS aggregation, while providing site-specific targeting (through binding to receptors on the cell surface and facilitating internalization). Further improvements in efficacy and therapeutic index of photoimmunoconjugates can be achieved by incorporating site-specific conjugation technologies such as engineered amino acids and peptide tags to reduce heterogeneity. Despite the fact that the development of the second-generation PS was started about 30 years ago, these technologies are still in demand and are in intensive development, especially in the direction of improving the process of optimizing linkers. Linkers of this type will increase the efficiency of the newly synthesized peptide/antibody-porphyrin conjugates, providing an efficient release of the beneficial properties of the PS at the target site.

The main limitations of using PS in first- and second-generation PDT include their insufficient solubility in water, long-term phototoxicity, and low efficiency on the tumor due to the limitation of the light penetration depth, whereas third-generation PS have a good response to various stimuli, such as temperature, pH and proteins. The main strategy for the development of third-generation photosensitizers is bioconjugation and encapsulation with targeting components based on precursors that is still at an early stage of research. A targeted drug delivery system with the effect of increased permeability and retention by tumor cells is one of the ultimate goals of the synthesis of such nanoparticles. Complexes for photoimmunotherapy have been developed. Specific antibodies are used for chemically bound to PS, which absorb light from the near infrared part of the spectrum. One of the immediate problems is to create nanoparticles with a photosensitizer, which should have an optimal wavelength for excitation of longer than 650 nm, allowing deeper penetration into the tissue. The significance for theranostics of dual-use conjugate systems should be noted, where radioactive, fluorescent, or photosensitizing molecules are simultaneously present. This will allow the use of such multifunctional conjugates for intraoperative radio detection, fluorescence imaging, and target PDT. Organometallic frameworks (MOFs) have been synthesized recently, where an inorganic building element (metal ions/clusters) is embedded in porphyrin molecules [85]. MOF plays an important role as a coadjuvant in PDT to alleviate hypoxia, reduce antioxidants, generate ROS, or act as a contrast agent for imaging therapy. Hyaluronic acid modified Chlorin e6 conjugated by encapsulation in one vessel and self-assembly with zeolite imidazolate framework-8 (ZIF-8@Ce6-HA) is an example of a PS delivery system based on MOF [86]. Such system demonstrates good biocompatibility, acceptable encapsulation efficiency, and is absorbed into cells. Moreover, the results of in vitro experiments demonstrated that ZIF-8@Ce6-HA exhibits high cytotoxicity after irradiation and leads to the death of about 88.4% of cells. Inductively coupled plasma mass spectrometry data showed that the modification of Ce6 HA increases the circulation time of ZIF-8@Ce6 in the blood and reduces its systemic toxicity. Organometallic scaffolds are classified as PS of the fourth generation, which indicates the prospects for the further development of such a promising method of PDT of tumors.

## 4. Conclusions

In this review, we briefly discussed the prospects of using new generations of porphyrin compounds for antitumor PDT, their synthesis, chemical characteristics, and role as diagnostic and therapeutic (theranostic) agents. Currently, in the molecular synthesis of effective PS approaches are used based on the modification of traditional tetrapyrrole structures, the creation of spin-orbit charge–transfer intersystem crossing (SOCT-ISC) or developments are underway in the direction of decreasing the singlet-triplet splitting (_Δ_EST) and thionation of carbonyl groups of fluorophores.

Over the past few years, most of the research in the field of second-generation PS synthesis has been carried out in the direction of the modification and optimization of porphyrins and related structures. Interest in the functionalization of PS to target molecules (antibodies, carbohydrates, amino acids, peptides, encapsulating carriers, liposomes, micelles and nanoparticle molecules) has increased significantly, leading to the development of third generation PS. It is possible to achieve this by interdisciplinary approaches that combine many technologies and form the basis for new conceptual applications. A similar synthesis of nanoscintillators by combining high-energy molecules and PS for X-ray PDT helps to solve the problem of limited light penetration into deep-seated cancers. Newly synthesized biomolecules have a pronounced effect of increased permeability, and when conjugated with suitable fragments for targeting and irradiated with a wavelength of more than 650 nm for deeper light penetration. Moreover, a PS should dissociate itself from its carriers and trigger photoreactions, which are determined by its physicochemical properties, and be rapidly metabolized and excreted from the body. The design and synthesis of such compounds should be carried out on the basis of molecular and oncological biology, using machine learning methods, which would allow PDT to be considered as a reference cancer treatment. On the other hand, the creation of new PS molecules with the desired pharmaceutical properties and their application in clinical trials are challenging tasks, which leaves newly synthesized third-generation PSs at the initial stage of research. The success of newly synthesized second-generation PSs based on biological components or conjugates with antibodies and receptor ligands in the field of theranostics is due to their properties. So, along with improved targeting ability, they have good biocompatibility and metabolic rate, which has an advantage over similar nanobase analogs, since such biomolecules do not create problems with elimination and safety during therapy. Nevertheless, the coordination of efforts in various fields of research in chemistry, biomedicine, biotechnology and bioengineering will open up new opportunities in the coming years and make PDT more promising.

## Data Availability

Not applicable.
